# The Essential Oil of *Salvia rosmarinus* Spenn. from Italy as a Source of Health-Promoting Compounds: Chemical Profile and Antioxidant and Cholinesterase Inhibitory Activity

**DOI:** 10.3390/plants9060798

**Published:** 2020-06-26

**Authors:** Mariarosaria Leporini, Marco Bonesi, Monica Rosa Loizzo, Nicodemo Giuseppe Passalacqua, Rosa Tundis

**Affiliations:** 1 Department of Pharmacy, Health and Nutritional Sciences, University of Calabria, 87036 Rende (CS), Italy; mariarosarialeporini@tiscali.it (M.L.); marco.bonesi@unical.it (M.B.); monica_rosa.loizzo@unical.it (M.R.L.); 2 Museum of Natural History of Calabria and Botanic Garden, University of Calabria, 87036 Rende (CS), Italy; nicodemo.passalacqua@unical.it

**Keywords:** *Salvia rosmarinus*, essential oil, GC, GC-MS, antioxidant potential, cholinesterase inhibitory activity

## Abstract

The chemical composition of the essential oil from *Salvia rosmarinus* Spenn. collected in Calabrian Ionian (R1) and Tyrrhenian (R2) coast (Southern Italy) was examined by gas chromatography (GC) and gas chromatography-mass spectrometry (GC-MS). Essential oils are mainly characterized by monoterpene hydrocarbons (39.32–40.70%) and oxygenated monoterpenes (36.08-39.47%). The 1,8-cineole, α-pinene, camphor, and *trans*-caryophyllene are the most representative compounds. *S. rosmarinus* essential oils were investigated for their antioxidant activity by using 2,2-diphenyl-1-picrylhydrazyl (DPPH), 2,2′-azino-bis (3-ethylbenzothiazoline-6-sulfonic acid) (ABTS), ferric reducing ability power (FRAP), and β-carotene bleaching tests. Additionally, acetylcholinesterase (AChE) and butyrylcholinesterase (BChE) inhibitory activity assays were used to screen the neuroprotective effects of *S. rosmarinus*. R2 showed the highest antioxidant potential as confirmed by relative antioxidant capacity index (RACI) and exhibited a selective activity against AChE (half maximal inhibitory concentration, IC_50_, value of 41.86 μg/mL). These results suggest *S. rosmarinus* essential oil as a potential source of bioactive compounds.

## 1. Introduction

*Salvia rosmarinus* Spenn., also known as rosemary, is a plant that belongs to the Lamiaceae family [1]. Rosemary is an evergreen, generally erect, rounded shrub with aromatic, needle-like, grey-green leaves and tiny, two-lipped, pale blue to white flowers. *S. rosmarinus* is native to dry scrub and rocky places in the Mediterranean areas of southern Europe to western Asia. Rosemary grows on loam soil with good drainage in an open, sunny position. It grows best in neutral to alkaline conditions with average fertility. *S. rosmarinus* is a well-known aromatic plant, used for thousands of years for ornamental, culinary, medicinal, and ritual proposes [2]. The most used name, *Rosmarinus officinalis* L., has to be considered a synonym of the actual name, *Salvia rosmarinus*, because molecular investigations evidenced as *Rosmarinus* L. is nested in *Salvia* L. [3]. The distribution area is manly in the western-central part of the Mediterranean basin, although in the eastern part it is considered introduced in several places [4]. *S. rosmarinus* has a remarkable morphological variability which led botanists to the taxonomic recognition of several specific and infraspecific taxa that are considered in the variability range of the species [5,6,7].

The analysis of the genetic variation pattern in the Mediterranean area [3] highlighted the presence of a basal haplotype from which four branches derived, consisting of a total of nine haplotypes clustered in two co-ancestry groups of populations. This work included one Calabrian population, which was found with the two widely shared basal haplotypes. Nonetheless, plants outside the core distribution of the species were proven to show a different chemical profile and biological activity [8]. Calabrian populations are in the eastern side of the species’ distribution range and differ from plants commonly cultivated by the prostrate habitus of individuals. 

Phytochemical studies on this *Salvia* species have reported the presence of different classes of bioactive compounds mainly including polyphenols, phenol diterpenes, and triterpenes. Rosmarinic acid, carnosic acid, carnosol, caffeic acid, betulinic acid, and ursolic acid are the dominant constituents. *S. rosmarinus* is also a rich source of essential oil [9]. Taking into account that numerous aspects, such as area of collection, time of harvest, and environmental and agronomic factors, influence the chemical composition of rosemary essential oil, several studies analysed the chemical profile of rosemary essential oil in different stages of ontogenesis in Greece [10] and Turkey [11], under the influence of different climatic factors in Tunisia [12,13,14,15] and Turkey [16], at different altitudes in Spain [17], and at different latitudes and longitudes in Sardinia (Italy) [18], as well as in dependence of pedological characteristics in France [19]. 

The essential oil of *S. rosmarinus* was demonstrated to possess antibacterial, antioxidant, antifungal, and anti-inflammatory properties [20]. Additionally, it is traditionally recognized to alleviate muscle pain and to improve cognitive diseases including Alzheimer disease (AD) [21]. AD is a neurodegenerative disease associated with loss of cholinergic neurons in parts of the brain and deposition of β-amyloid in the form of neurofibrillary tangles and amyloid plaques [22,23]. At present, there is no a therapeutic approach that can delay the AD progression, but available treatment has provided symptomatic benefits. Nowadays, two main classes of drugs are available for AD patients: Cholinesterase inhibitors, such as donepezil, galantamine, and rivastigmine, and the glutamate antagonist memantine [24]. Two cholinesterases, acetylcholinesterase (AChE) and butyrylcholinesterase (BChE), participate in cholinergic neurotransmission by hydrolysing acetylcholine (Ach) in the central and peripheral nervous system. AChE is responsible for the degradation of Ach in the synaptic cleft of cholinergic synapses and neuromuscular junctions into choline and acetate. It has a crucial role in regulating many functions such as learning, memory, cerebral blood flow control, and cortical organization of movement. On the other hand, BChE has a higher activity in liver, heart, intestine, kidney, and lung. The enzymes share 65% amino acids’ sequence homology and show similar molecular forms and active sites. Oxidative stress has been identified as a contributing factor in the progression of neurodegenerative diseases. The abnormal cellular metabolism caused by the excessive production of reactive oxygen species (ROS) could affect both production and accumulation of β-amyloid that could exacerbate cellular dysfunction and ROS production, thus contributing to a vicious cycle [25]. Thus, restoring the level of acetylcholine and the use of natural antioxidants may be proposed in the treatment and management of AD [26,27]. 

Following our previous studies [28,29,30], the present work aimed to analyse the chemical composition, the antioxidant effects, and the anti-cholinesterase activities of Italian *S. rosmarinus* essential oils. The chemical profile, the antioxidant properties, and acetylcholinesterase (AChE) and butyrylcholinesterase (BChE) inhibitory activity have been investigated.

## 2. Results and Discussion

### 2.1. Chemical Profile 

The essential oils obtained by hydrodistillation of *S. rosmarinus* aerial parts collected in two areas of Calabria (Southern Italy) were herein investigated. The main constituents are listed in Table 1. Thirty-one compounds were identified, accounting for 97.48 and 98.78% of the total composition of essential oil for R1 (essential oil of *S. rosmarinus* from Ionian coast) and R2 (essential oil of *S. rosmarinus* from Tyrrhenian coast), respectively. Chromatograms are reported in Supplementary Materials (Figures S1 and S2).

The main volatiles were monoterpene hydrocarbons (40.7 and 39.32%, respectively per R1 and R2), followed by oxygenated monoterpenes (36.08 and 39.47%, respectively per R1 and R2) and sesquiterpene hydrocarbons (16.5 and 18.21%, respectively per R1 and R2). The 1,8-cineole, α-pinene, *trans*-caryophyllene, and β-pinene are the most abundant compounds of both rosemary essential oils (Figure S3). The 1,8-cineole content was highest in R2 (21.89%) compared to R1 (16.98%). *Trans*-caryophyllene content was greater in R1 (10.58) than R2 (8.62%). Conversely, a similar value was observed for α-pinene (10.96 and 10.37%, for R2 and R1, respectively). Significant amounts of camphor (11.08 and 7.27%, respectively for R2 and R1) and camphene (6.87% and 6.30%, respectively for R2 and R1) were found. The δ-3-sarene, α-cubebene, δ-selinene, β-bisabolene, and γ-cadinene were not detected in R1 sample. On the other hand, linalool, α-thujone, terpinen-4-ol, and α-copaene were not identified or were absent in sample R2.

The relative quantities, presence, and/or absence of rosemary essential oils’ constituents are strongly affected by environmental conditions and agronomic management practices, as shown in literature data. According to Pintore et al. [31], Angioni et al. [10] demonstrated that Sardinian rosemary essential oil is an α-pinene/borneol/bornyl acetate/verbenone chemotype. Compared with our essential oils, Sardinian samples presented more content in α-pinene (~23%), borneol (~16%), bornyl-acetate (~10.4%), and camphene (~7.6%) but lower camphor content in camphor (~4.5%). Verbenone compound was not detected in our essential oils. In Corsican rosemary essential oil, the same trend and similar values were observed: α-pinene > borneol > bornyl acetate > verbenone. 

Forty-one samples of *S. rosmarinus* collected in different locations of Sicily were analysed by Napoli et al. [32]. Monoterpenes, both hydrocarbons (range of 21–68%) and oxygenated (range of 29–79%), were the most highly represented components. Three essential oils obtained from aerial parts of *S. rosmarinus* growing in Tunisia were analysed with detection of 38 components. Among them, 1,8-cineole and camphor were found in more content (33.08–37.75% and 13.55–18.13%, respectively) than our essential oils, but similar values for α-pinene (8.58–9.32%), α-terpineol (6.79–8.17%), camphene (5.07–5.58%), and borneol (4.08–5.48%) were observed [33].

The different chemical composition of the essential oils is subject to change under the influence of various factors including climatic conditions, environmental factors, and time of collection [34]. 

### 2.2. Cholinesterase Activity

In order to examine the in vitro neuroprotective effects of rosemary essential oils, the inhibitory activity of acetylcholinesterase (AChE) and butyrylcholinesterase (BChE) enzymes was assessed.

Indeed, in spite of the multi-factorial nature of AD, the available therapeutic approach currently applied is based on the cholinergic hypothesis, which postulates that at least some of the cognitive decline experienced by patients affected by AD results from a deficiency in neurotransmitter acetylcholine and, thus, in a reduced cholinergic neurotransmission in brain cortical or hippocampal regions. Therefore, restoring the levels of acetylcholine through the inhibition of both cholinesterases is a useful therapeutic approach to treat AD. Both rosemary essential oils inhibited AChE and BChE in a concentration-dependent manner. The half maximal inhibitory concentration (IC_50_) values and selectivity index (SI) are reported in Table 2. R2 sample exhibited a good inhibitory activity against AChE (IC_50_ of 41.86 μg/mL), 2.1-times higher in comparison to R1 (IC_50_ of 85.96 μg/mL).

Interestingly, R2 Italian essential oil showed stronger activity against AChE enzyme compared to other rosemary essential oils previously investigated. Ben Jemia et al. [20] confirmed the neuroprotective activity of *S. rosmarinus* essential oil from Tunisia. In this study, eight essential oils were screened and the best results were observed for samples collected in Matmata and Dj. Khamess with IC_50_ value of 64.7 against AChE. 

A promising AChE inhibitory activity was observed also with Spanish *S. rosmarinus* essential oil with IC_50_ values of 68.4 μg/mL [35]. The results are in accord with Mata et al. [36] who assessed the AChE inhibitory activity of rosemary essential oil from Portugal, finding an IC_50_ of 69.8 μg/mL. Less activity was reported for Tunisian *S. rosmarinus* essential oil with inhibition percentage of 36.2% at concentration of 50 mg/mL against AChE [37]. Turkish *S. rosmarinus* essential oil was previously investigated by Orhan et al. [38]. An inhibitions’ percentage of 63.7 and 74% at concentration of 1 mg/mL, respectively for AChE and BChE enzymes, was observed. 

Among identified compounds, α-pinene, 1,8-cineole, and camphor exhibited IC_50_ values of 0.63, 0.67 mM, and >10 mM and showed to be competitively reversible inhibitors of AChE [39]. More recently, 1,8-cineole and camphor were investigated against AChE. IC_50_ values of 2.27 and 21.43 µM, respectively, were found [40]. A percentage of AChE inhibition of 48.5% at 1 mM was found for β-pinene [41]. In another work, *trans*-caryophyllene revealed an AChE inhibition of 32% at the final concentration of 0.06 mM with a more selective activity against BChE (IC_50_ value of 78.6 μM) [21].

Some studies have analysed structure–activity relationships for monoterpenes and AChE inhibitory activity [42]. One of these works proposed that the interactions of the hydrocarbon skeletal of terpenes are with the AChE hydrophobic active center. Since the hydrophobicity degree of the active site on the AChE is described to be different among the globular forms of AChE, this may further explain the variation in inhibition seen between studies. In conclusion, the inhibitory activity of the essential oil likely results from a complex interaction of its chemical components, ultimately producing synergistic or antagonistic inhibitory responses [20,43]. Indeed, essential oils are mixtures of components that exhibit generally higher activities than their pure constituents. Their final activities are due to the combined effects of components.

### 2.3. Antioxidant Activity

Literature data evidenced a correlation between oxidative stress and degenerative diseases. Indeed, the oxidative stress is a key hallmark of generation and accumulation of ROS that promote neuronal cell death [17]. Additionally, an elevated level of lipid peroxidation marker in the brain of AD patients, especially in the region of the temporal lobe, was found [44]. Iron-induced oxidative stress, as evidenced by iron accumulation in the brain of AD, is responsible for neurodegeneration in patients diagnosed with AD. This iron accumulation in the brain of AD patients leads to the formation of hydroxyl radicals through the Fenton reaction [44]. The search of new cholinesterases’ inhibitors capable of exerting antioxidant effects is, therefore, desirable.

Herein, in order to estimate the antioxidant potential effects, *S. rosmarinus* essential oils were investigated by using different in vitro assays, namely 2,2-diphenyl-1-picrylhydrazyl (DPPH), 2,2′-azino-bis(3-ethylbenzothiazoline-6-sulfonic acid) (ABTS), ferric reducing ability power (FRAP), and β-carotene bleaching test (Table 3). An approach based on the use of different tests is required because antioxidants can exert their capacity through different mechanisms of radicals’ inactivation [45]. This scientific evidence may justify the diversity of results in relation to the applied test.

In DPPH test, R1 sample exhibited a greater radicals’ scavenging activity with a percentage of 33.21%. Conversely, the ABTS^+^ radical cation was more sensible to R2 oil that showed an IC_50_ value of 22.61 μg/mL. This evidence can be justified because ABTS^+^ and DPPH radicals possessed a different stereochemistry and a different training mechanism and, after reaction with essential oils, these two radicals can produce different responses.

In the β-carotene bleaching test after 30 min of incubation, R2 demonstrated the highest capacity to inhibit lipid peroxidation (IC_50_ of 33.18 μg/mL). The ability to induce the reduction of iron ion, measured with FRAP test, was greater for R2 with value of 5.59 μM Fe (II)/g.

Our results differed from those recently reported by Chraibi et al. [46] who analysed the radical scavenging power against DPPH of *S. rosmarinus* essential oil from Morocco and found a better activity with IC_50_ value of 2.77 mg/mL. El Kamli et al. [47] reported the potential of Moroccan rosemary essential oils to inhibit lipid peroxidation. However, all samples demonstrated less antioxidant capacity compared to our samples, showing IC_50_ values in the range of 18.12–24.23 mg/mL. Previously, Bouyahya et al. [48] assessed the antioxidant activity of Moroccan *S. rosmarinus* essential oils but, in comparison with our results, this essential oil showed major activity with IC_50_ value of 85.74 and 523.41 μg/mL, respectively, for FRAP and DPPH test. The antioxidant ability of essential oils obtained from seven Iranian populations of *S. rosmarinus* was analysed, finding lower inhibition percentages ranging from 28.73–73.69%, tested at concentration of 3.6 mg/mL in DPPH test [49].

Greater antioxidant activity was previously reported for rosemary, investigated at three different stages of development, collected near Lake Garda (Italy) [50]. The authors compared the essential oils’ radical scavenging capacity at flowering, post-flowering, and vegetative stage. The best activity was observed at flowering stage with IC_50_ values of 36.78 μL, followed by post-flowering, and vegetative state (79.69 and 111.94 μg/mL, respectively). 

Fifteen essential oils from the aerial parts of Algerian *S. rosmarinus* were investigated by Hendel et al. [51]. All the samples showed moderate antioxidant activity, being the half-maximal scavenging concentration (SC_50_) values in the range of 120.4–326.1 μL/mL. The antioxidant activity of *S. rosmarinus* essential oil from France was confirmed by Miladi et al. [52] who found a greater activity with IC_50_ value of 189 μg/mL in DPPH test.

Previously, Kadri et al. [53] screened the *S. rosmarinus* essential oil from Tunisia for its in vitro antioxidant activities using DPPH, β-carotene bleaching test, and FRAP assay. The results of the DPPH assay showed an IC_50_ value of 110.20 μg/mL, better than our results. In the β-carotene bleaching test, Tunisian essential oil showed a higher inhibition of lipid peroxidation (IC_50_ of 27.28 μg/mL). The half maximal effective concentration (EC_50_) (concentration at which the absorbance is 0.5) value of *S. rosmarinus* essential oil was 38.68 μg/mL. Interestingly, Hussain et al. [54] reported that essential oil of *S. rosmarinus*, cultivated in Pakistan, showed strong antioxidant activity (IC_50_ of 20.9 μg/mL). 

**Figure 1:**
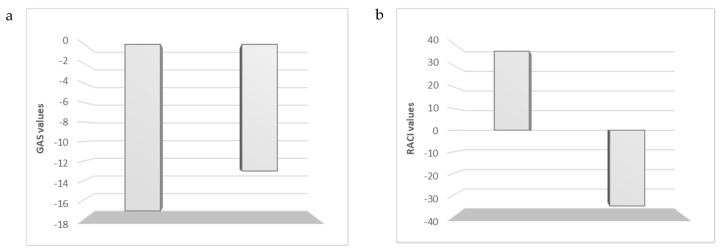
Global antioxidant score (GAS) (**a**) and Relative antioxidant capacity index (RACI); (**b**) values of rosemary essential oils.

Relative antioxidant capacity index (RACI) and global antioxidant score (GAS) analyses were applied to evaluate the antioxidant potential of essential oils (Figure 1). Based on RACI (36.06 and −35.11 for R1 and R2, respectively) and GAS data (−17.14 and −13.04 for R1 and R2, respectively), *S. rosmarinus* essential oil from the Tyrrhenian coast was more active than that from the Ionian coast.

Nie et al. [55] evaluated the radical scavenging activity of active compounds of rosemary essential oil in DPPH and ABTS test. In the first test, the active order was camphor > bornyl acetate > *p*-cymene > 3-carene > *o*-cymene > α-pinene > terpinen-4-ol > camphene > linalool oxide acetate > β-pinene > α-bisabolene. The authors suggested that the carbonyl group is important to free radical scavenging activity and the presence of a double bond conjugated to a carbonyl group will further enhance the antioxidant activity. In ABTS test, the active order was *o*-cymene > camphene > α-pinene > camphor > bornyl acetate > α-bisabolene. It was observed that camphor and bornyl acetate showed strong activity in scavenging DPPH radical but less activity against ABTS radical cation due to different kinds of free radicals and the way antioxidants interact with free radicals. Indeed, both affected the scavenging effect of antioxidants. In this case, the cyclic ether group was particularly important for ABTS radical cation scavenging. Thus, the antioxidant activity was correlated to essential oil composition and, consequently, to oxygenated monoterpenes and mixture of mono- and sesquiterpene hydrocarbons [33]. In addition, according to Bajalan et al. [49], the antioxidant activity was positively correlated with the most abundant compounds, such as β-pinene or 1,8-cineole. In a previous study, 1,8-cineole and α-pinene demonstrated to prevent lipid peroxidation [56]. Comparing both compounds, α-pinene showed the major capacity to inhibit lipid peroxidation. The higher lipophilic character of this monoterpene drives strong interactions with cell membranes. Interestingly, α-pinene and 1,8-cineole increased the activity and protein expression of the main antioxidant enzymes including catalase, glutathione peroxidase, glutathione reductase, and superoxide dismutase. However, it is important to know that minor compounds may act in synergism with the most abundant compounds.

## 3. Materials and Methods 

### 3.1. Chemicals and Reagents

Solvents of analytical grade were obtained from VWR International s.r.l. (Milan, Italy). Ascorbic acid, propyl gallate, butylated hydroxytoluene (BHT), 2,2-azinobis(3-ethylbenzothiazoline-6-sulfonic) acid (ABTS) solution, 2,2-diphenyl-1-picrylhydrazyl (DPPH), tripyridyltriazine (TPTZ), β-carotene, Tween 20, linoleic acid, acetylcholinesterase (AChE) from *Electrophorus electricus* (EC 3.1.1.7, Type VI-S) and butyrylcholinesterase (BChE) from equine serum (EC 3.1.1.8), acetylthiocholine iodide (ATCI), butyrylthiocholine iodide (BTCI), 5,5′-dithio-bis(2-nitrobenzoic acid) (DTNB), and physostigmine were purchased from Sigma-Aldrich S.p.a. (Milan, Italy).

### 3.2. Plant Materials

*S. rosmarinus* were collected in Calabria (Southern Italy) in Cirò Marina, Ionian coast [R1 (Crotone, latitude: 39°22′43″ N, longitude: 17°01′52″ E, 360 m above the sea), voucher specimen n. CLU 23969], and in Praia a Mare, Tyrrhenian coast [R2 (Cosenza, latitude: 39°52′14″ N, longitude: 15°47′26″ E, 30 m above the sea), voucher specimen n. CLU 23974]. Both sites are characterized by a Mediterranean climate. However, the R1 site results with a dry-thermo-Mediterranean bioclimate, whereas in the R2 site the bioclimate is sub humid-meso-Mediterranean. Plants of rosemary grow in a shrubby habitat with an open canopy structure and some bare ground (garrigue) on a rocky limestone soil. 

Aerial parts of a single plant per each locality were harvested in order to obtain an adequate quantity for the analysis. Plant materials were examined for integrity and absence of dust and insect contamination. The authentication was carried out by Dr. Nicodemo G. Passalacqua at the Natural History Museum of Calabria and the Botanic Garden, University of Calabria (Italy).

### 3.3. Extraction Procedure

Fresh *S. rosmarinus* areal parts (0.74 and 1.20 kg for R1 and R2, respectively) were subject to hydrodistillation for 3 h using a Clevenger-type apparatus [57]. The white-yellow, obtained essential oils (3.21 and 7.01 mL) were dried over anhydrous sodium sulphate, stored in hermetically sealed brown glass bottles, and kept at 4 °C before analysis. Yields (w/w) of 0.58 and 0.41% for R1 and R2, respectively, were obtained.

### 3.4. Gas Chromatography (GC) and Gas Chromatography-Mass Spectrometry (GC-MS) Analyses

The chemical composition of *S. rosmarinus* essential oils was screened by gas chromatography associated with mass spectrometry, using a Hewlett-Packard gas chromatograph (Agilent, Milan, Italy) equipped with a non-polar HP-5 capillary column (30 m × 0.25 mm, 0.25 μm), associated with a Hewlett-Packard mass spectrometer (Agilent, Milan, Italy). The ionization of the sample constituents was performed in electronic impact (EI, 70 electronvolt). The analyses were carried out with the following temperature schedule: Isotherm at 50 °C for 5 min, temperature increase from 50 to 250 °C of 5 °C/min, and finally isotherm at 250 °C for 10 min. Helium was used as carrier gas (1.0 mL/min). One μL of diluted essential oil (1/10 v/v, in *n*-hexane) was injected (split ratio 1:20). Essential oils were also analyzed using a Shimadzu GC17A gas chromatograph, equipped with an ionization flame detector (FID) and an HP-5 capillary column (30 m × 0.25 mm, 0.25 μm) (Shimadzu, Milan, Italy). Nitrogen was used as transport gas. The conditions used were the same as those described for the GC-MS analyses. Retention index were determined in relation to a homologous series of *n*-alkanes (C_8_-C_24_) under the same operating conditions. 

The identification of compounds was based on the comparison of their RI, either with those in literature or with those of available authentic standards (Sigma-Aldrich, Milan, Italy), on the comparison of the mass spectral data with the Wiley 138 library, and referring to the spectral data of pure compounds.

### 3.5. In Vitro Anti-Cholinesterase Activity

The inhibition of AChE and BChE enzymes was measured by using a modified colorimetric Ellman’s method [20] based on the reaction of released thiocholine to give a colored product with a chromogenic reagent such as AChE from *E. electricus* (EC 3.1.1.7, Type VI-S) and BChE equine serum (EC 3.1.1.8). Acetylthiocholine (ATCI) iodide and butyrylthiocholine iodide (BTCI) were employed as the substrates of the reaction. In brief, enzyme, essential oils, and phosphate buffer were mixed in microplates and incubated in an ice bath at 4 °C. After 30 min, physostigmine was added. The reaction started by adding 5,5’-dithiobis(2-nitrobenzoic-acid) (DTNB) solution and substrate. The microplate was placed in a thermostatic water bath (Branson model 3800-CPXH, Milan, Italy) for 20 min at 37 °C. The reaction was stopped by placing the microplate in an ice bath and adding physostigmine. The absorbance was measured at 405 nm. The percentage inhibition was calculated by the equation: 


Inhibition (%) = [(Blank − Blank positive control) − (Experiment − Experiment control)]/[Blank − Blank positive control] × 100.

Physostigmine was used as positive control. Results are calculated as IC_50_ values (μg/mL).

### 3.6. In Vitro Antioxidant Activity

Four in vitro assays, namely DPPH, ABTS, FRAP, and β-carotene bleaching tests, were herein applied to investigate the antioxidant effects of R1 and R2 essential oils. Often natural antioxidants may be multifunctional and may act in vivo through different mechanisms. Therefore, no single technique can completely evaluate the antioxidant potential of a plant extract.

#### 3.6.1. DPPH and ABTS Tests

The radical scavenging potential was examined using two different spectrophotometric methods, such as DPPH and ABTS tests. 

DPPH assay was performed according to the procedure previously reported [58]. The DPPH solution (1.0 × 10^−4^ M) and rosemary essential oils at different concentrations (62.5–1000 μg/mL) were mixed. After 30 min, the absorbance was measured at 517 nm.

The DPPH radicals’ scavenging activity was calculated by using the following equation: DPPH radicals’ scavenging activity (%) = [1 − (sample absorbance with DPPH − sample absorbance without DPPH)] × 100. ABTS test was employed following the procedure previously used by [59]. ABTS solution (7 mM) and potassium persulphate (2.45 mM) were mixed in order to obtain ABTS radical cation solution (ABTS^+^). After 12 h, ABTS^+^ was diluted with ethanol to final absorbance of 0.70 at 734 nm. Successively, 2 mL of diluted ABTS^+^ solution was added to extracts (25 μL) at concentrations (1–400 μg/mL). After 6 min the absorbance was read at 734 nm. The ABTS scavenging capacity was calculated following the equation: ABTS radicals’ scavenging activity (%) = [(A_0_ − A)/A_0_] × 100, where A_0_ is the absorbance of the control reaction and A is the absorbance in the presence of extract. 

In both tests, ascorbic acid was used as positive control. Results are calculated as IC_50_ values (μg/mL).

#### 3.6.2. FRAP Assay

The ability to reduce iron ions was assessed using FRAP test [58]. A solution of tripyridyltriazine (TPTZ, 10 mM), 2.5 mL of FeCl_30_ (20 mM), HCl (40 mM), and 25 mL of acetate buffer (0.3 M) at pH 3.6 was prepared in order to obtain FRAP reagent. The latter (2.0 mL), water (900 μL), and essential oil (100 μL) at a concentration of 2.5 mg/mL were mixed. After 30 min of incubation, the absorbance was measured at 595 nm. Butylated hydroxytoluene (BHT) was used as a positive control. The FRAP value was expressed as μM Fe (II)/g. 

#### 3.6.3. β-Carotene Bleaching Test

The ability of rosemary essential oils to protect lipid peroxidation was assessed by using the β-carotene bleaching test [59]. Propyl gallate was the positive control. In brief, a mixture of β-carotene, linoleic acid, and 100% Tween 20 was prepared. The obtained emulsion was added to a 96-well microplate containing sample at concentrations ranging from 100 to 2.5 μg/mL. The absorbance was measured at 470 nm against a blank at t = 0 and after 30 and 60 min of incubation. The antioxidant activity was calculated as follows: AA = [(A_0_ − A_t_) /(A_0_* − A_t_*)] × 100, where A_0_ and A_0_* are the absorbance values obtained at the time 0 for samples and control, respectively, while A_t_ and A_t_* are the absorbance values obtained after 30 and 60 min of incubation for samples and control, respectively.

### 3.7. Statistical Analysis

All experiments were performed in triplicate (*n* = 3). Data are expressed as means ± standard deviation (SD). The concentration giving 50% inhibition (IC_50_) was calculated by nonlinear regression with the use of Prism GraphPad Prism version 4.0 for Windows (GraphPad Software, San Diego, CA, USA). The concentration-response curve was obtained by plotting the percentage inhibition versus concentration. Differences within and between groups were evaluated by one-way analysis of variance test (ANOVA) followed by a multi-comparison Dunnett’s test that was used to compare each group with the positive control in biological assays. Differences were considered to be significant at *p* < 0.05 in the biological test. Relative antioxidant capacity index (RACI) is an integrated statistical application to analyse the antioxidant capacity values generated by different in vitro methods [60].

Data obtained from antioxidant assays were employed to calculate RACI value by using the following equation: RACI = (x − μ)/σ, where x is the raw data, μ is the mean, and σ is the standard deviation. Moreover, for each sample, the average of T-scores was used to calculate global antioxidant score (GAS) value [60].

## 4. Conclusions

In this work, two Calabrian rosemary populations (collected on the Ionian and Tyrrhenian coasts) were studied. Chemical analyses revealed 1,8-cineole, α-pinene, camphor, and *trans*-caryophyllene as the most representative compounds. However, differences occurred between samples, suggesting that a different chemical composition of the essential oils is subject to change under the influence of various factors including climatic conditions and environmental factors. Our results confirmed *S. rosmarinus* essential oils, in particular, the Tyrrhenian sample, as a promising source of natural cholinesterase inhibitors useful in the management and treatment of neurodegenerative diseases.

Obtained data provide the basis for further in vivo studies in order to establish synergistic and antagonistic effects, and the pharmacokinetic parameters, such as dose and administration route, that could corroborate these first results on the potential health benefits of *S. rosmarinus* essential oils and their pure constituents. 

## Figures and Tables

**Table 1:** The main constituents (%) of *Salvia rosmarinus* essential oils.

Nr.	Compound	Class	RI ^a^	R1	R2	I.M ^b^	Sign
1	Thujene	MH	926	2.34 ± 0.03 ^a^	0.88 ± 0.05 ^b^	1,2	**
2	α-Pinene	MH	938	10.37 ± 0.01 ^a^	10.96 ± 1.33 ^b^	1,2,3	**
3	Camphene	MH	953	6.30 ± 0.24 ^a^	6.87 ± 0.21 ^b^	1,2,3	**
6	Sabinene	MH	973	2.82 ± 0.11 ^a^	1.01 ± 0.06 ^b^	1,2,3	**
4	β-Pinene	MH	980	7.89 ± 1.03 ^b^	8.23 ± 0.62 ^a^	1,2,3	**
5	Myrcene	MH	993	1.32 ± 0.06 ^b^	2.73 ± 0.12 ^a^	1,2,3	**
7	α-Phellandrene	MH	1005	0.70 ± 0.02 ^a^	0.39 ± 0.04 ^b^	1,2	**
8	δ-3-Carene	MH	1009	nd	0.34 ± 0.02 ^a^	1,2	**
9	α-Terpinene	MH	1012	1.19 ± 0.08 ^b^	1.35 ± 0.03 ^a^	1,2,3	**
10	*o*-Cymene	MH	1020	0.25 ± 0.2 ^a^	tr	1,2	**
11	*p*-Cymene	MH	1025	1.80 ± 0.18 ^a^	0.67 ± 0.07 ^b^	1,2	**
12	Limonene	MH	1030	1.78 ± 0.06 ^b^	2.30 ± 0.09 ^a^	1,2,3	**
13	1,8-Cineole	OM	1034	16.98 ± 2.11 ^b^	21.89 ± 2.32 ^a^	1,2,3	**
14	γ-Terpinene	MH	1057	2.76 ± 0.25 ^a^	2.42 ± 0.06 ^b^	1,2,3	**
15	Terpinolene	MH	1086	1.18 ± 0.05 ^a^	1.17 ± 0.01 ^a^	1,2,3	ns
16	Linalool	OM	1098	0.35 ± 0.06 ^a^	nd	1,2,3	**
17	α-Thujone	OM	1106	0.10 ± 0.01 ^a^	nd	1,2	**
18	Camphor	OM	1145	7.27 ± 0.23 ^b^	11.08 ± 0.76 ^a^	1,2	**
19	Borneol	OM	1167	5.30 ± 0.26 ^a^	3.31 ± 0.08 ^b^	1,2	**
20	Terpinen-4-ol	OM	1176	2.03 ± 0.09 ^a^	nd	1,2	**
21	α-Terpineol	OM	1189	4.05 ± 0.26 ^a^	3.19 ± 0.10 ^b^	1,2,3	**
22	(-)-Bornyl acetate	SH	1286	2.40 ± 0.11 ^b^	4.26 ± 0.12 ^a^	1,2	**
23	α-Cubebene	SH	1352	nd	0.43 ± 0.03 ^a^	1,2	**
24	α-Copaene	SH	1377	0.24 ± 0.03 ^a^	nd	1,2	**
25	*trans*-Caryophyllene	SH	1415	10.58 ± 1.98 ^a^	8.62 ± 0.17 ^b^	1,2,3	**
26	Aromadendrene	SH	1437	0.30 ± 0.03 ^a^	0.25 ± 0.04 ^a^	1,2	ns
27	α-Humulene	SH	1455	1.95 ± 0.05 ^a^	1.49 ± 0.10 ^b^	1,2	**
28	γ-Muurolene	SH	1478	0.32 ± 0.05 ^a^	0.32 ± 0.01 ^a^	1,2	ns
29	α-Amorphene	SH	1487	0.25 ± 0.02 ^a^	tr	1,2	**
30	δ-Selinene	SH	1493	tr	0.58 ± 0.02 ^a^	1,2	**
31	β-Bisabolene	SH	1508	tr	0.41 ± 0.01 ^a^	1,2	**
32	γ-Cadinene	SH	1515	tr	0.44 ± 0.05 ^a^	1,2	**
33	δ-Cadinene	SH	1526	0.46 ± 0.03 ^b^	1.41 ± 0.01 ^a^	1,2	**
34	Caryophyllene oxide	OS	1580	0.65 ± 0.04 ^a^	0.54 ± 0.02 ^b^	1,2	**
35	Viridiflorol	OS	1591	1.65 ± 0.07 ^a^	tr	1,2	**
36	Manool	OS	2055	1.90 ± 0.08 ^a^	1.24 ± 0.06 ^b^	1,2	**
	MH			40.70	39.32		
	OM			36.08	39.47		
	SH			16.50	18.21		
	OS			4.20	1.78		
	Total identified			97.48	98.78		

R1: *S. rosmarinus* from Ionian coast; R2: *S. rosmarinus* from Tyrrhenian coast. Monoterpene hydrocarbons: MH; oxygenated monoterpenes: OM; sesquiterpene hydrocarbons: SH; oxygenated sesquiterpenes: OS. Data are reported as the mean ± standard deviation (*n* = 3). ^a^ RI: Retention indices on the HP 5MS (5%-phenyl)-methylpolysiloxane nonpolar column. ^b^ IM, identification method: 1. Comparison of retention times, 2. Comparison of mass spectra with MS libraries, 3. Comparison with authentic compounds; tr: Trace (<0.1%); nd: Not detected. Differences were evaluated by one-way analysis of variance (ANOVA) completed with a multicomparison Tukey’s test; ** *p* < 0.05. Means in the same row with different small letters differ significantly (*p* < 0.05). Sign: Significant; ns: Not significant.

**Table 2:** Cholinesterases’ inhibitory activity of rosemary essential oils.

Sample	AChE IC_50_ (µg/mL)	BChE IC_50_ (µg/mL)	SI (BChE/AChE)
R1	85.96 ± 3.12 ^****^	46.71 ± 1.85 ^****^	0.54
R2	41.86 ± 1.63 ^****^	48.29 ± 1.90 ^****^	1.15
*Positive control*			
Physostigmine	0.12 ± 0.01	0.21 ± 0.03	2.0

Data are expressed as means ± S.D (*n* = 3). SI: selective index. Differences within and between groups were evaluated by one-way ANOVA followed by a multicomparison Dunnett’s test α = 0.05): **** *p* < 0.0001 compared with the positive control.

**Table 3:** In Vitro antioxidant activity of rosemary essential oils.

Sample	DPPH Test ^a^ IC_50_ (µg/mL)	ABTS Test IC_50_ (µg/mL)	β-carotene Bleaching Test IC_50_ (µg/mL)		FRAP Test μM Fe (II)/g
R1	33.21%	35.43 ± 2.83 ^****^	45.21 ± 2.76 ^****^	49.48 ± 3.08 ^****^	2.95 ± 1.46 ^****^
R2	29.84%	22.61 ± 1.91 ^****^	33.18 ± 2.11 ^****^	44.81 ± 2.75 ^****^	5.59 ± 1.95 ^****^
*Positive control*					
Ascorbic acid	5.02 ± 0.80	1.70 ± 0.06			
Propyl gallate			0.09 ± 0.004	0.09 ± 0.004	
BHT					63.22 ± 4.3

Data are expressed as means ± S.D (*n* = 3). ^a^ At concentration of 1000 μg/mL. Differences within and between groups were evaluated by one-way ANOVA followed by a multicomparison Dunnett’s test α = 0.05): **** *p* < 0.0001 compared with the positive controls.

## Supplementary Materials

The following are available online at https://www.mdpi.com/2223-7747/9/6/798/s1, Figure S1: Chromatogram of *S. rosmarinus* essential oil from Ionian coast (sample R1), Figure S2: Chromatogram of *S. rosmarinus* essential oil from Tyrrhenian coast (sample R2), Figure S3: Mass spectra of the most representative constituents of rosemary essential oil.
