# Supplementary material for: The Essential Oil of Salvia rosmarinus Spenn. from Italy as a Source of Health-Promoting Compounds: Chemical Profile and Antioxidant and Cholinesterase Inhibitory Activity
Source: Plants (Basel). 2020 Jun 26;9(6):798. doi: 10.3390/plants9060798 (PMC7356759; doi:10.3390/plants9060798)
Supplement: Supplementary file 1 [file plants-09-00798-s001.pdf]

SUPPLEMENTARY MATERIALS

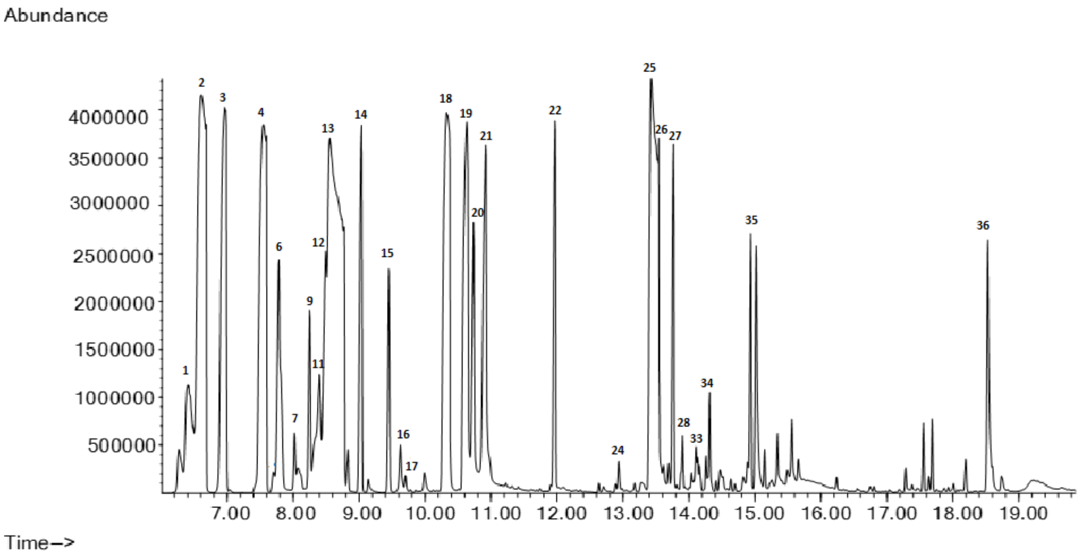

Figure S1. Chromatogram of *S. rosmarinus* essential oil from Ionian coast (sample R1). 1. Thujene (Retention time, Rt: 6.40 min); 2. α-Pinene (Rt: 6.60 min); 3. Camphene (Rt: 6.96 min); 4. β-Pinene (Rt: 7.75); 6. Sabinene (RT: 7.78); 7. α-Phellandrene (RT: 8.02 min); 9. α-Terpinene (Rt: 8.24 min); 11. *p*-Cymene (Rt: 8.40 min); 12. Limonene (Rt: 18.47 min); 13. 1,8-Cineole (Rt: 8.56 min); 14. γ-Terpinene (Rt: 9.03 min); 15. Terpinolene (Rt: 9.44 min); 16. Linalool (RT: 9.62 min); 17. α-Thujone (RT: 9.70 min); 18. Camphor (Rt: 10.33 min); 19. Borneol (Rt: 10.63 min); 20. Terpinen-4-ol (Rt: 10.73 min); 21. α-Terpineol (Rt: 10.91 min); 22. (-)-Bornyl acetate (Rt: 11.96 min); 24. α-Copaene (Rt: 12.93); 25. *trans*-Caryophyllene (Rt: 13.43 min); 26. Aromadendrene (Rt: 13.51 min); 27. α-Humulene (Rt: 13.76 min); 28. γ-Muurolene (Rt: 13.90 min); 33. δ-Cadinene (Rt: 14.31 min); 34. Caryophyllene oxide (Rt: 14.47 min); 35. Viridiflorol (Rt: 15.02 min); 36. Manool (RT: 18.53 min).

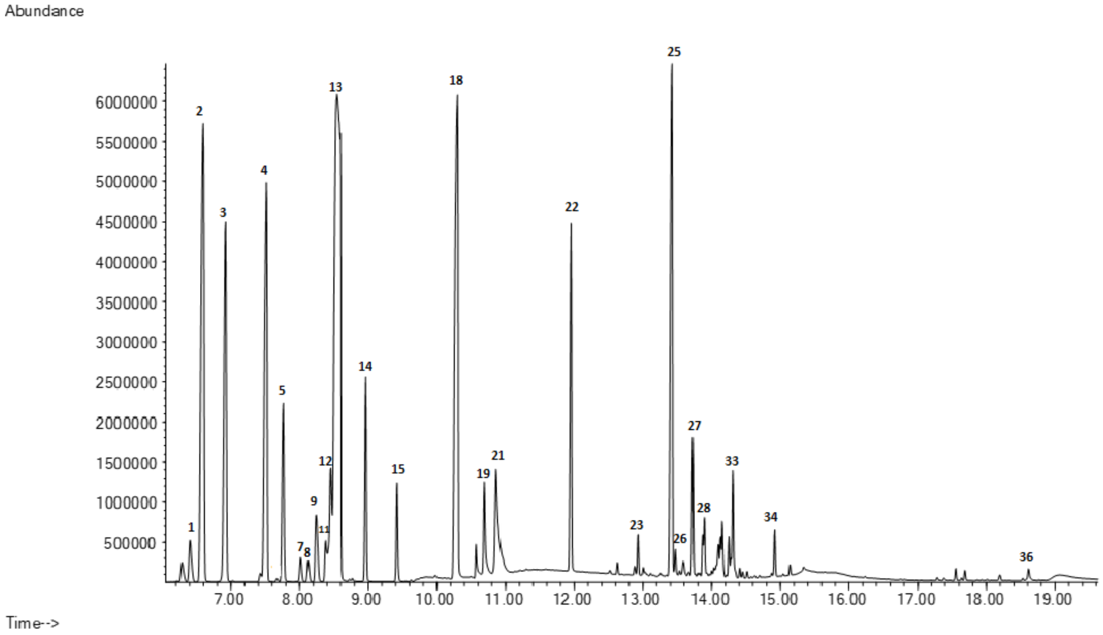

Figure S2. Chromatogram of *S. rosmarinus* essential oil from Tyrrhenian coast (sample R2). 1. Thujene (Retention time, Rt: 6.40 min); 2. α-Pinene (Rt: 6.59 min); 3. Camphene (Rt: 6.92 min); 4. β-Pinene (Rt: 7.51); 5. Myrcene (Rt: 7.76 min); 7. α-Phellandrene (Rt: 8.02 min); 9. α-Terpinene (Rt: 8.24 min); 11. *p*-Cymene (Rt: 8.38 min); 12. Limonene (Rt: 18.45 min); 13. 1,8-Cineole (Rt: 8.54 min); 14. γ-Terpinene (Rt: 8.96 min); 15. Terpinolene (Rt: 9.42 min); 18. Camphor (Rt: 10.30 min); 19. Borneol (Rt: 10.68 min); 21. α-Terpineol (Rt: 10.86 min); 22. (-)-Bornyl acetate (Rt: 11.95 min); 23. α-Cubebene (Rt: 12.93); 25. *trans*-Caryophyllene (Rt: 13.42 min); 26. Aromadendrene (Rt: 13.45 min); 27. α-Humulene (Rt: 13.72 min); 28. γ-Muurolene (Rt: 13.87 min); 33. δ-Cadinene (Rt: 14.31 min); 34. Caryophyllene oxide (Rt: 14.91 min); 36. Manool (Rt: 18.51 min).

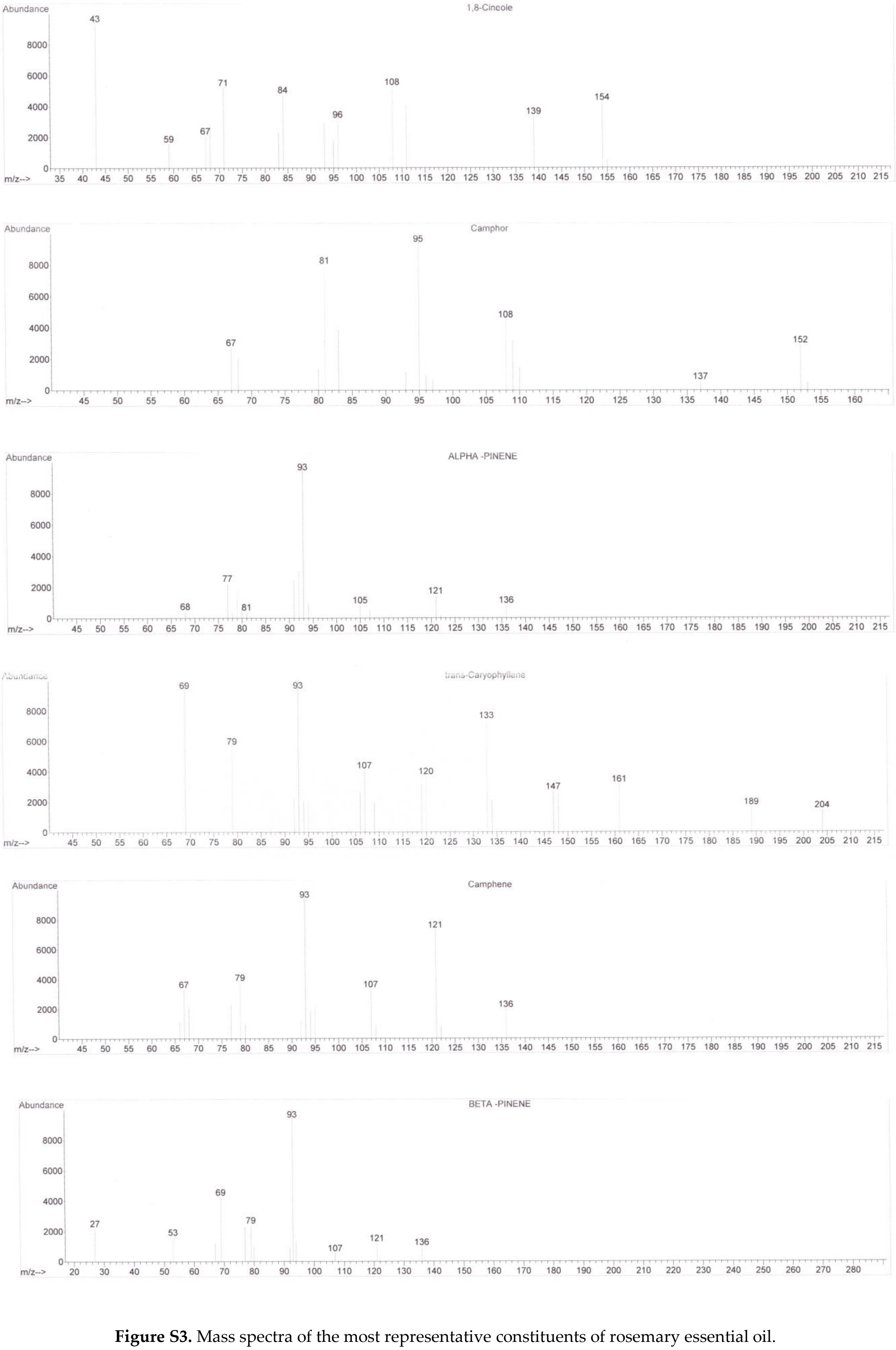

Figure S3. Mass spectra of the most representative constituents of rosemary essential oil.
